# Life-long Programming Implications of Exposure to Tobacco Smoking and Nicotine Before and Soon After Birth: Evidence for Altered Lung Development

**DOI:** 10.3390/ijerph8030875

**Published:** 2011-03-16

**Authors:** Gert S. Maritz, Richard Harding

**Affiliations:** 1 Department of Medical Biosciences, University of the Western Cape, Bellville 7535, South Africa; E-Mail: gmaritz@uwc.ac.za; 2 Department of Anatomy and Developmental Biology, Monash University, Clayton, VIC 3800, Australia

**Keywords:** lung structure, lung function, nicotine, metabolism, alveoli, conducting airways

## Abstract

Tobacco smoking during pregnancy remains common, especially in indigenous communities, and likely contributes to respiratory illness in exposed offspring. It is now well established that components of tobacco smoke, notably nicotine, can affect multiple organs in the fetus and newborn, potentially with life-long consequences. Recent studies have shown that nicotine can permanently affect the developing lung such that its final structure and function are adversely affected; these changes can increase the risk of respiratory illness and accelerate the decline in lung function with age. In this review we discuss the impact of maternal smoking on the lungs and consider the evidence that smoking can have life-long, programming consequences for exposed offspring. Exposure to maternal tobacco smoking and nicotine intake during pregnancy and lactation changes the genetic program that controls the development and aging of the lungs of the offspring. Changes in the conducting airways and alveoli reduce lung function in exposed offspring, rendering the lungs more susceptible to obstructive lung disease and accelerating lung aging. Although it is generally accepted that prevention of maternal smoking during pregnancy and lactation is essential, current knowledge of the effects of nicotine on lung development does not support the use of nicotine replacement therapy in this group.

## 1. Introduction

Recent epidemiological studies have shown that lung function and susceptibility to respiratory diseases throughout life can be programmed by environmental factors operating during fetal and early postnatal life. One of the most common factors that can result in reduced lung function and respiratory health is exposure to maternal tobacco smoking, both before and after birth. Other factors include poor nutrition (both maternal and neonatal), maternal alcohol consumption, intra-uterine infections, early postnatal infections and exposure to allergens. For all of these, the timing of exposure in relation to lung development, as well as the level of exposure, will determine the severity of the effects on later lung function and respiratory illness. In this review we will focus on the immediate and long-term (or programming) effects of maternal tobacco smoking on lung development and respiratory function. As nicotine is a major component of tobacco smoke and has been identified as a risk factor for diseases in infants and children [1,2] we will emphasize the programming effects of early exposure to nicotine. In addition to effects on the lungs, nicotine exposure via the mother may affect the development of multiple organs; for example it has been shown to have long-lasting effects on body adiposity and endocrine function of offspring [3].

Although smoking during pregnancy is the leading cause of fetal morbidity and mortality and obstetric disease, many pregnant women continue to smoke. In North America 20–25% of pregnant women smoke tobacco and in Spain this figure varies between 30% and 36% [4]. Although nearly 41% of smokers try to quit the habit each year, relapse is common, and only about 10% achieve and maintain abstinence. The unpleasant effects of nicotine withdrawal account in part for the low success rate. Approved pharmacotherapies to treat nicotine dependence, such as nicotine replacement therapy (NRT) and Buproprion [5], have moderate efficacy. Thus additional and more effective therapies are required [6]. Varenicline appears to be one such therapy [7], provided that the benefits outweigh the risks to the mother and offspring.

## 2. Effects of Maternal Tobacco Smoking on Lung Function and Respiratory Health of Offspring

Prenatal and early postnatal exposure to tobacco smoke has a wide range of adverse health effects, including an increased risk of low birthweight and perinatal complications, the Sudden Infant Death Syndrome (SIDS), obstructive lung disease, altered neurodevelopment and childhood infections and cancers [8]. Arguably the most common of these is the adverse effect on lung function and respiratory health of perinatally exposed infants and children, and even adults. Clinical and epidemiological data from people whose mothers smoked tobacco have collectively provided strong evidence that exposure to the components of tobacco smoke during gestation and/or infancy can alter lung development such that later lung function and respiratory health are impaired. In infants prenatally exposed to maternal smoking, tidal and forced expiratory flow rates are reduced, suggesting that small airway development has been affected [9,10]. This is supported by studies in guinea pigs which showed that maternal smoke exposure during pregnancy affected structural and functional development of the small conducting airways of the offspring soon after birth [11]. It is likely that changes in airway structure and function that are present in infancy persist until childhood and potentially into adulthood. Numerous studies over the last 20 years have shown that children who are exposed to tobacco smoke during gestation are at increased risk of having airway hyperresponsiveness and asthma [12]. Prenatal smoke exposure in particular has been associated with reduced lung function in children, implicating restricted airflow in small conducting airways [13]. The effects of early smoke exposure on airway hyperresponsiveness appear to continue until at least early adulthood, suggesting that effects of smoke exposure on the small airways are permanent [14]. A potential mechanism for reduced lung function is a reduction in the number of alveolar-bronchiolar attachment points, due to reduced alveolarization. It is possible that maternal smoking could affect lung development by increasing oxidative stress in the lungs. However, the hypothesis that polymorphisms in maternal anti-oxidant genes could play a role in the adverse influence of maternal smoking on lung function in children was not supported by a recent study [15].

There is some evidence that lung function in adults could be affected by perinatal smoke exposure, independent of personal lifestyle [8,16]. Maternal smoking may also increase the risk of low FEV_1_ and COPD in adults [17], but it is presently unclear whether such effects are due to prenatal smoking or exposure to environmental tobacco smoke during infancy and childhood.

## 3. Nicotine Uptake

Nicotine is arguably the major physiologically active component of tobacco smoke and is rapidly absorbed from the respiratory tract of smokers. Although it has often been assumed that pulmonary absorption of nicotine from inhaled cigarette smoke is more rapid than by other routes (e.g., oral and transcutaneous), the lung appears to serve as a reservoir for nicotine, which slows its entry into the arterial circulation [18]. This implies that rather than all of the nicotine inhaled in each puff being absorbed in a few seconds, it may require 30–60 seconds or longer for the nicotine to be absorbed. Once in the maternal circulation, nicotine readily crosses the placenta and enters the fetal circulation [19]; it can enter the amniotic fluid and from there it can be absorbed via the skin of the fetus [20]. Nicotine enters breast milk, and can reach concentrations that are approximately 2–3 times that in maternal plasma. This is primarily due to the partitioning of nicotine into the high-lipid-containing [21], more acidic milk [22,23].

The typical American smoker who consumes 17 cigarettes per day absorbs systemically about 0.3 mg nicotine/kg body weight per day [24]. Blood or plasma nicotine concentrations sampled in the afternoon in cigarette smokers generally range from 10 to 50 ng/mL [25]. The tissues with the highest affinity for nicotine are the liver, kidney, spleen and lungs; the lowest affinity for nicotine is in adipose tissue. It also binds with high affinity to brain tissue [25,26].

When a pregnant woman smokes tobacco, nicotine enters the fetal circulation via the placenta. Although nicotine readily crosses the placenta there is no evidence that it is metabolized by the placenta. It is therefore likely that the blood concentrations of nicotine reached in the fetus are similar to those in the mother; however, there is no direct evidence supporting the notion. Peak nicotine levels in the pregnant mother’s blood occur 15–30 minutes after it is administered [27]. Most of the nicotine that enters the fetus returns to the maternal circulation for elimination, although some enters the amniotic fluid via the fetal urine. Consequently nicotine and cotinine accumulate in the amniotic fluid of the pregnant smoker because the nicotine eliminated by the fetus is added to the nicotine coming from the blood vessels of the amniochorionic membrane [23]. The fetus is therefore likely to be exposed to nicotine even after concentrations in maternal blood have decreased.

## 4. Metabolism of Nicotine during Pregnancy

The clearance of nicotine and cotinine, the major product of nicotine metabolism, is increased in pregnant women [28]. This can be ascribed to an increase in liver blood flow and an increased enzymatic breakdown of nicotine and cotinine in the mother. Since the enzymatic protection mechanisms of the fetus are not well developed [29–31], the metabolism of nicotine in the fetal liver is slow and a longer half-life of nicotine in the fetus can be expected. This is confirmed by the higher concentrations of nicotine in fetal tissue compared to maternal blood levels [32]. Consequently the cells of the developing lung and other organs are exposed to higher concentrations of nicotine for longer periods of time and thus to the adverse effects of nicotine on cell integrity. This is important as nicotine is genotoxic [33] and induces the release of oxidants [34]. Since rapidly dividing cells are more vulnerable to the effects of foreign substances such as nicotine [35], it is conceivable that nicotine exposure during gestation and early postnatal life via maternal milk may interfere with growth and development. This can be achieved in two ways: by having a direct effect on cells and/or by reducing the nutrient supply to the fetus during gestation and lactation. It has been shown that long-term nicotine exposure results in a predisposition for genetic instability [21,36,37]. This may result in changes in the genetic “program” that controls lung development, maintenance of lung structure and aging of lung tissue, which may render the lungs more prone to disease.

## 5. Nicotine and Oxidant/Antioxidant Status

It has been shown that maternal smoking is associated with increased levels of oxidative stress markers in the mother and offspring [38,39]. There is also convincing *in vivo* and *in vitro* evidence suggesting that exposure to nicotine results in oxidative stress in fetal, neonatal and adult tissues [39,40]. Reactive oxygen species (ROS) target mitochondria, and mitochondrial DNA has been shown to be more sensitive to the deleterious effects of ROS than nuclear DNA [41]. In addition, the electron transport chain enzyme complexes in the inner membrane of the mitochondria are extremely sensitive to ROS inactivation [42].

In addition to inducing overproduction of oxidants, nicotine exposure results in a decrease in the activity of SOD and catalase. It also results in a decrease in the levels of low molecular weight antioxidants such as vitamins C and E [43]. Along with the decrease in the antioxidant capacity of the body, concentrations of malondialdehyde (MDA) are increased, indicating oxidant damage to the cells [1,2]. The increase in ROS levels, together with a decrease in the activities of enzymes with antioxidant function, results in an imbalance in the oxidant/antioxidant capacity. This imbalance is maintained long after nicotine withdrawal [2] and becomes worse with age [34].

It is conceivable that the increased levels of nicotine-induced ROS in the fetus and suckling neonate as a consequence of maternal smoking or NRT will result in not only mitochondrial DNA damage but also damage of nuclear DNA. It is therefore likely that nicotine and ROS will result in a change in the capacity of the mitochondria to deliver energy and to participate in homeostatic mechanisms and in changing the “program” that controls growth, tissue maintenance, aging and cellular metabolism.

## 6. Effects of Maternal Nicotine on Nutritional, Hormonal and Biochemical Profiles in the Offspring

A number of studies indicate that some women who quit smoking during gestation relapse again during lactation. Lactation is a sensitive period during which cognitive and neurologic developments occur in suckling offspring. In a recent study it was shown that maternal nicotine intake, only during the period of lactation, leads to long-term effects on body weight (BW) regulation, leptin concentration, and thyroid function in adult rat offspring [44]. In rat experiments it has been shown that, when neonates were exposed to nicotine in milk during suckling, their circulating catecholamine concentrations were higher than those of controls. After weaning, catecholamine levels decreased to normal but it is possible that the transient early adrenal medullary dysfunction caused by nicotine exposure may have a later impact on cardiovascular control in adult progeny [3].

## 7. Nicotine-Induced Body Malformations

It is believed that the early period of organogenesis is the most vulnerable stage of embryogenesis to environmental insults [32]. Changing the *in utero* environment during early organogenesis may impair the process and in this way alter the structure and function of organs in the long term. Tobacco smoke introduces more than 4,000 chemicals into the circulation. Many of these chemicals, including nicotine, cross the placental barrier and enter the blood of the developing embryo and fetus. They can also enter the amniotic fluid and in this way alter the environment within which the embryo and fetus grows and develops. Nicotine is a major teratogenic component of tobacco smoke which can perturb embryogenesis. Studies in rats have shown that nicotine can induce embryonic abnormalities, such as neural tube malformations, before and during the early stages of organogenesis, in a concentration-dependent manner [45]. The nicotine-induced embryonic malformations were associated with increases in programmed cell death in embryos. Nicotine can also cause cell death by increasing intracellular calcium levels and oxidative stress in the embryo [45]. Severe embryonic malformation may result in embryonic demise and is associated with higher spontaneous abortion and miscarriage in humans [46]. These malformations are thought to be caused by the nicotine-induced overproduction of ROS [45].

Animal studies show that a variety of antioxidants are effective in decreasing the damaging effects of heightened oxidative stress induced by teratogens. Effective antioxidants which might also be of clinical value include vitamins C and E, carotenoids, folic acid, as well as synthetic products. Appropriate clinical studies with antioxidants in pregnancies at high risk of developing oxidative stress are needed, since non-toxic antioxidants might prove an efficient and inexpensive way to reduce the rate of some serious and potentially fatal congenital anomalies [47].

## 8. Effect of Nicotine on the Development of the Lung

There is growing evidence that nicotine which is transported across the placenta may be the key constituent of cigarette smoke that alters lung development in offspring, thereby leading to impaired lung function and an increased risk of respiratory illness. After entering the fetal circulation, nicotine interacts with nicotinic acetylcholine receptors (nAChRs) in the fetal lung. This causes changes in lung structure and function in the offspring. Consistent with this, it has been shown that α3, α5, and α7 nicotinic acetylcholine receptors (nAChR) are expressed in non-neuronal cells in the lungs of fetal monkeys, and that maternal nicotine exposure up-regulates nAChR expression in fetal lung [48]. High affinity nAChRs are found in the membranes of normal lung cells and in lung cancer cells of all histological types [49–51]. These include α3, α5, α7, and β2 or β4 subunits [50], of which α7 may help to modulate cell shape and affect cell-to-cell contact. It has been demonstrated that nicotine promotes cell proliferation upon its interaction with nAChRs on the surface of rodent bronchial epithelium and may contribute to dysanaptic lung growth [52].

Fetal exposure to nicotine has been shown to reduce the surface complexity of the lung parenchyma, increase collagen accumulation, up-regulate surfactant protein gene expression, and induce neuro-endocrine cell hyperplasia in fetal lungs; collectively these changes alter pulmonary function [53]. In addition it has been shown that non-neuronal cells in lung synthesize acetylcholine (ACh), and that a cholinergic autocrine loop exists in developing lung [54]. Thus, prenatal nicotine exposure likely affects lung development by modifying the actions of this autocrine cholinergic loop. Much remains, however, to be determined about the mechanism by which nicotinic signaling alters lung development.

Nicotine also activates several cellular pro-survival signals [55,56]. An example is the increase in the activity of protein kinase C (PKC) in various human and murine lung cancer cell lines when exposed to nicotine. Nicotine also elicits the activity of Raf-1 [57]. The activation of these kinases has been shown to be responsible for the phosphorylation of Bcl-2 which antagonizes opioid-induced apoptotic signaling in lung cancer cells [49,58,59]. An increase in the phosphorylation of Akt has been detected *in vivo* in the lungs of nicotine-treated mice and in human lung cancer cells derived from smokers. The activation of this kinase is associated with tobacco-related carcinogenesis in the lung. The activation of these kinases in cultured cells that were transiently exposed to nicotine, suggests that nicotine directly or indirectly contributes to the process of lung carcinogenesis [49,55,59,60].

It has also been shown that long-term nicotine exposure results in a predisposition for the induction of genetic instability [36,37,61]. Gene amplification is a hallmark of gene instability. Gene instability requires two critical elements, namely an inappropriate cell cycle progression, and DNA damage. Long-term nicotine exposure, through the activation of Ras pathways and up regulation of cyclin D1, disrupts the G1 arrest. It also augments the production of ROS which may lead to DNA damage. This implies that exposure to nicotine via tobacco smoke or via NRT will make the lungs more prone to the development of cancer [36].

## 9. Effects of Nicotine on Metabolic Activity in the Lung

### 9.1. Energy Metabolism

Glucose uptake and metabolism are essential for the proliferation and survival of cells, and may be enhanced in actively proliferating cell systems such as embryonic tissue. Glucose is considered to be an essential source of energy in lung tissue [62] and is necessary for the functional development of the lung [63–65]. Glucose is also the main source of α-glycerophosphate for pulmonary surfactant synthesis in the adult lung while in fetal lung, the loss of cellular glycogen from alveolar type II cells just before birth is associated with increased surfactant synthesis [66]. During the alveolar phase of lung development, which occurs from around week 36 of gestation in humans [67], lung tissue is more dependent on glycogen as an energy substrate than adult lung. This is illustrated by the fact that during fasting the phosphorylase activity of adult lung tissue decreases to conserve glycogen while it increases in fetal and neonatal lung, thereby increasing the utilization of the lung glycogen stores. This means that the control of glycogen metabolism during the alveolar phase of lung development is different from that of adults [68].

Although glucose and glycogen are the primary energy substrates of adult and developing lung, fatty acids are also important. For example, during fasting, when blood fatty acid levels are elevated, fatty acids replace glucose as the primary energy substrate. Under these circumstances glucose is conserved by the lung for α-glycerophosphate synthesis and eventual surfactant formation by the type II alveolar epithelial cells [69].

**Figure 1.**
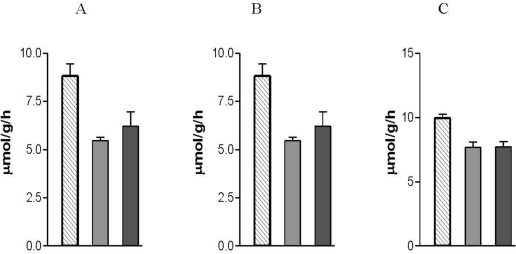
The influence of maternal nicotine exposure during gestation and lactation on: (A) glucose (Control *vs.* Experimental groups: P < 0.001), (B) glycogen utilization (Control *vs.* Experimental groups: P < 0.001) and (C) lactate production (Control *vs.* Experimental groups: P < 0.01) by lung tissue of the offspring. Hatched bars show data from Control offspring (postnatal days 21 and 42 data combined); Grey bars show data from Nicotine exposed offspring killed at postnatal day 21; Black bars show data from a Withdrawal group at postnatal day 42. In the Withdrawal group, nicotine was not available from weaning on postnatal age 21 until the animals were killed at day 42; a period of 21 days of nicotine withdrawal was allowed to establish the longer term effect of nicotine exposure on carbohydrate metabolism by lung tissue of the offspring. Age-matched Control tissue was used in each case [68,71]. Data are presented as mean ± SEM.

Nicotine exposure during gestation and the early postnatal period results in sustained suppression of glycogenolysis and glycolysis in lung tissue (Figure 1) [70,71]. The lower glycogenolytic activity is due to a lower phosphorylase activity in the lungs of nicotine exposed offspring [71]. The ratio of inactive to active phosphorylase of lung tissue of nicotine exposed offspring is the same as for animals that were not exposed to nicotine via the placenta and mother’s milk. However, the tissue levels of both phosphorylase fractions are lower than in the lungs of the control animals, which implies that the total phosphorylase content of the lungs of the nicotine exposed animals was lower than that of the control animals. This means that maternal nicotine exposure suppresses the synthesis of phosphorylase in the lungs of the offspring. It also implies that maternal nicotine exposure had no direct inhibitory effect on the activity of the phosphorylase in the lungs of the offspring. The lower rate of glycogen breakdown in the lungs of animals that were exposed to nicotine via the placenta and mother’s milk was due to a permanent lower glycogenolytic activity. The implication is that the developing fetal and neonatal lungs of these animals are more dependent on exogenous glucose for utilization via the hexose monophosphate shunt than on glucose derived from the lung’s glycogen stores [71].

The uptake of exogenous glucose is carried out by glucose transporters. Glucose transporter isoforms 1 (Glut 1) and 4 (Glut 4) are not present in adult lung, but are present in developing lung [72]. Over-expression of these Glut isoforms can enhance glucose uptake into fetal lungs to support active cell proliferation, which is a common characteristic of developing lung epithelium [73]. The decrease in the flux of glucose through the glycolytic pathway of lungs of nicotine exposed rat pups is, however, not due to compromised glucose transporters because the total glucose turnover of the lung tissue of rats that were exposed to nicotine via the placenta and mother’s milk is higher than in animals that were not exposed to nicotine. The higher glucose flux is actually due to a faster utilization of glucose via the hexose monophosphate shunt [74]. After nicotine withdrawal the flux of glucose through the glycolytic pathway remained suppressed to the same degree than while exposed to nicotine (Figure 1). After nicotine withdrawal, the flux of glucose through the hexose monophosphate pathway returns to normal [70]. In addition to the reduced flux of glucose through the glycolytic pathway [70], AMP accumulates in the lungs of the nicotine exposed rat pups [75].

**Figure 2.**
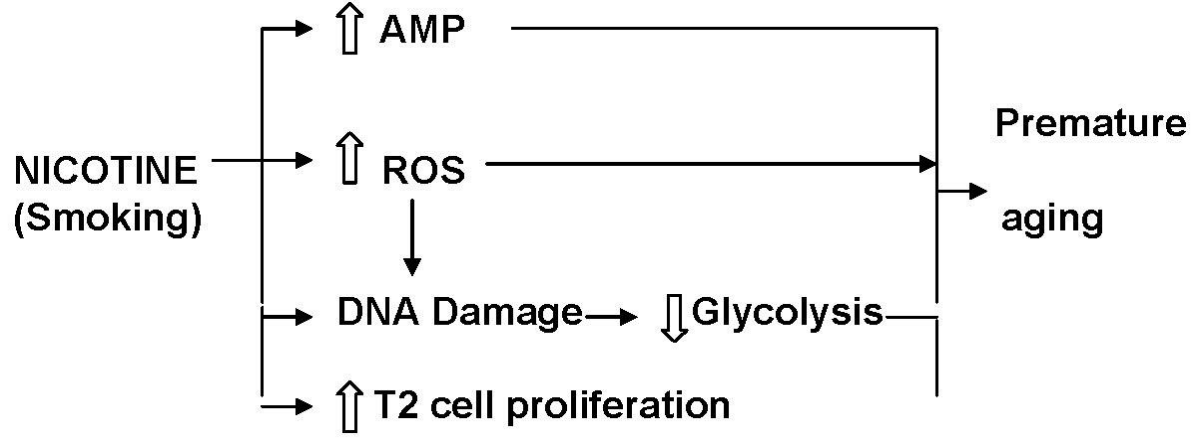
Diagram to illustrate the factors that induce premature aging of the lung parenchyma of rats exposed to nicotine via tobacco smoke or nicotine replacement therapy (NRT) via the placenta and mother’s milk. Premature aging of the lung is associated with an increased propensity for emphysema.

Both the persistent reduction in glycolytic activity and high levels of AMP are associated with premature onset of cell senescence [76,77]. This is supported by studies showing that enhancement of glycolysis prevents cellular senescence [78]. It is, therefore, conceivable that nicotine exposure during gestation and suckling induces premature aging of the lungs by irreversible suppression of glycolysis and the persistent high levels of AMP in the lungs of the offspring (Figure 2).

### 9.2. Xenobiotic Metabolism

The respiratory system is one of the major targets for exposure to exogenous substances [79]. A major source of exogenous chemicals to which the respiratory system is exposed is inhaled tobacco smoke [80]. In addition to exposure to air-borne substances the respiratory system is also exposed to chemicals via the systemic circulation [81]. This is particularly true during gestation when the developing lungs are exposed to chemicals transferred to the fetus from the maternal circulation, and during lactation when compounds are conveyed to the newborn via the mother’s milk [82]. As nicotine freely crosses the placenta [83] and occurs in significant quantities in the milk of smoking mothers [82], it can interact with the developing fetus and neonate of mothers who either actively smoke tobacco or use NRT.

It is widely accepted that the cytochrome P450 (CYP) superfamily of enzymes is the principal means by which the lung metabolizes exogenous substances [79]. Upon entering the lung many of the chemicals are not hazardous as such, but are frequently biotransformed by CYP enzymes into reactive intermediates. A critical factor contributing to the etiology or modification of respiratory disease is whether the lung tissue has the ability to activate or efficiently inactivate chemicals [83]. Recent studies have indeed shown that maternal nicotine exposure during gestation and lactation results in a permanently elevated expression of CYP2A3 and CYP 2B1 [84]. It has been shown that CYP2A6 plays an important role in the formation of cancer-inducing agents such as 4-(methylnitrosoamino)-(3-pyridyl)-1-butanone (NNK). It is also known that the rat orthologue of CYP2A3 induces the synthesis of NNK [85]. The permanent increase in expression of these CYPs may thus increase the susceptibility of the lungs of nicotine exposed offspring to cancer.

The Mn-Zn SOD activity of the developing lungs is also reduced by nicotine exposure which further reduces the ability of the lungs to be protected against the point mutations in DNA induced by oxidants; this will increase the susceptibility of exposed lungs to changes in the “program” that controls lung development, maintenance and aging.

## 10. Effects of Nicotine on Structural Development of the Lungs

Fibroblasts play a critical role in the transition from the saccular to the alveolar stage of lung development, during which there is a four-fold increase in the number of interstitial fibroblasts in the neonatal rat lung [86]. Perturbations such as hyperoxia, barotrauma and steroid therapy have been shown to interfere with alveolar development in the rat [87], baboon [88] and human [89], the net result of which is a significant, often permanent, decrease in the number of alveoli.

Although the control of alveolar formation is poorly understood, a substantial body of evidence exists regarding events that coincide with alveolar septation, many of which may influence fibroblast proliferation. Elastic fibers are thought to be involved in septation by providing structural support for newly emerging secondary septa. Inhibition of elastic fiber assembly has been linked to impaired septation and alveolarization [90]. In neonatal rat lung fibroblasts, elastin expression peaks during the second postnatal week, that is during the phase of rapid alveolarization, and declines rapidly thereafter [91]. This means that interference with lung fibroblast integrity may result in impaired formation of alveoli, which may lead to a permanent reduction in the number of alveoli.

**Figure 3.**
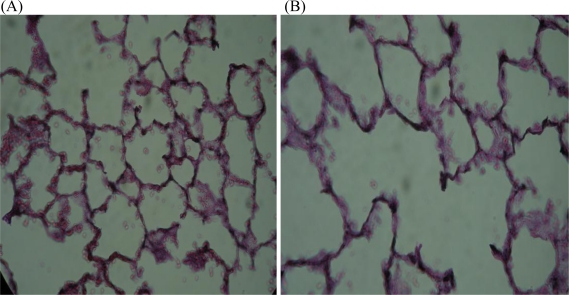
Effect of maternal nicotine exposure during pregnancy and lactation in rats on the connective tissue framework (stained black) of the lung of adult offspring. Note that the connective tissue framework of the control lungs (A) is more extensive than that of the offspring that were exposed to nicotine via the mother (B) [102].

Exposure to cigarette smoke inhibits fibroblast proliferation and migration by increasing cell cycle transit time, thereby reducing the rate of alveolarization [92]. Consequently the surface area available for gas exchange is reduced; another effect of reduced alveolarization is a reduction in the number of alveolar-bronchiolar attachments, which can lead to airway narrowing [93]. Cigarette smoke exposure also compromises fibroblast-induced repair responses, and may be one of the factors that contributes to the development of smoke-induced lung diseases [94]. Accumulation of nicotine in fibroblasts will affect glycolysis and plausibly fibroblast function too. However, *in vitro* studies have shown that nicotine has no effect on fibroblasts from human fetal lungs [94]. *In vivo* studies also show that nicotine only has a transient effect on metabolism in lungs of adult animals, as opposed to a permanent suppression of energy metabolism of animals that were exposed to nicotine during lung development [75]. The *in vitro* studies on fibroblasts were performed on cells that were not metabolically permanently compromised as opposed to the fibroblasts of lung cells of neonatal rats that had been exposed to nicotine during gestation and lactation. Therefore, since nicotine exposure during gestation and lactation interferes with glucose metabolism and apoptosis in the fetal and neonatal lung, and since it may cause disruption of the interaction between lung fibroblast glucose metabolism and fibroblast function, it is plausible that it will also adversely affect the long-term maintenance of lung structure. It is interesting to note that lung fibroblasts from patients with emphysema show a reduced proliferation rate [95] and premature aging [96] and that this condition is characterized by slow degeneration of the lung parenchyma [97]. Therefore, the gradual deterioration of the connective tissue framework of the lungs of nicotine exposed rat pups (Figure 3) may be partially due to inadequate fibroblast proliferation and function.

Many agents that induce lung injury may do so by modifying key metabolic events for various cell populations in the lung. Type I alveolar epithelial cells for example, which cover more than 90% of the alveolar surface [98], depend on glycolysis for energy [99]. Glycolysis also supplies the ATP required to maintain the membrane-linked Na + -K + ATPase [100]. The Na + -K + ATPase pump plays a vital role in maintaining cell volume. Therefore, reducing its activity by inhibition of glycolysis will result in the swelling of these cells and the formation of membrane “blebs” [101].

**Figure 4.**
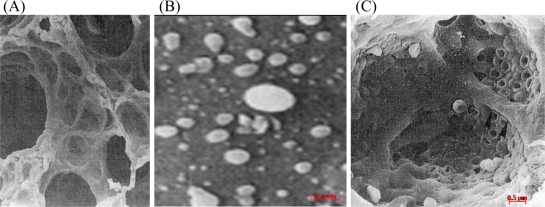
Scanning electron micrographs of the alveolar surface in postnatal rats showing (A) control lung, (B) blebbing of the alveolar type I cell membrane in a nicotine exposed animal and (C) rupture of the alveolar surface to reveal the underlying capillary filled with red blood cells in a nicotine exposed animal. The nicotine exposed rats received nicotine during gestation and lactation. All animals were sacrificed on postnatal day 21 and lung tissue processed for scanning electron microscopy [102].

Inhibition of glycolysis would therefore be expected to interfere with the ability of the type I alveolar epithelial cells to adapt to changes in the environment and to maintain cell volume. Since pulmonary glycolysis is irreversibly suppressed in animals that were exposed to nicotine during lung development, the activity of the Na + -K + ATPase pump will also be permanently lower in type I epithelial cells, and this could result in membrane blebbing and rupture of the cell membranes (Figure 4). The type I epithelial cells are the most vulnerable to injury [102] and the permanently reduced glycolytic activity will therefore make them even more susceptible to damage, especially when exposed to toxic substances in blood and inhaled air.

Alveolar type II cell proliferation has been found to be increased in the lungs of nicotine exposed animals, which is a likely response to type I cell injury and death [103]. Type II alveolar epithelial cells are critical for the maintenance of alveolar homeostasis by secreting surfactant and by proliferating and differentiating to replace damaged type I cells [104]; in effect these cells act in defense of the alveolus. It has also been shown that maternal nicotine exposure during gestation and lactation induces rapid type II alveolar epithelial cell proliferation in response to type I cell damage [105–107]. Since rapid cell proliferation is associated with rapid shortening of the telomeres [108], it is conceivable that premature aging of the type II cells will occur in the lungs of the nicotine exposed rats. This may result in an increased vulnerability of the alveolus, which is supported by the observation that loss of type II cells has a detrimental effect on the alveolus [106].

**Figure 5.**
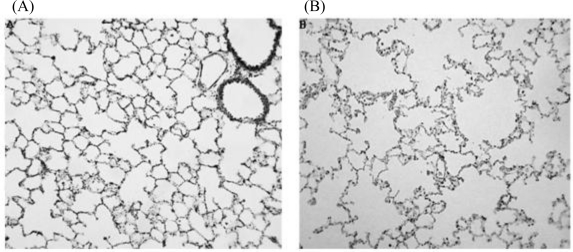
The effect of nicotine exposure via the mother during pregnancy and lactation on the parenchyma of the lung tissue of adult offspring. The alveoli of the control lungs (A) are smaller than those of the lungs that were exposed to nicotine (B).

It appears that the negative impact of maternal nicotine exposure during gestation and lactation on the growth, development and repair processes of the lungs of the offspring causes lung structure to more rapidly deteriorate with age than in animals that were not exposed to nicotine. This is illustrated by the appearance of membrane blebs (Figure 4), alveolar fenestrations [109] and eventually microscopic emphysema (Figure 5). The elastic tissue framework (Figure 3) of the lungs of nicotine exposed animals is also compromised [110]. Exposure of fetal monkeys and rats to nicotine via the placenta during the late saccular/early alveolar phase of lung development results in an increase in the size of the primitive alveoli; as a consequence the alveolar surface area for gas exchange in the adult lung is decreased [109,111]. Collectively, the structural changes in the lungs of these animals resembles faster aging of the lungs, and are likely to make the lungs more susceptible to respiratory disease.

The gradual deterioration of the lung parenchyma with increasing age is clearly due to an inability of the lung epithelium and fibroblasts to maintain the structural integrity of the lungs. This effect is likely due to premature aging of the fibroblasts [96] and alveolar epithelial cells [112], which can be attributed to altered “programming” due to the changes in the *in utero* environment [111,113,114].

The reason for the altered “programming” is not clear. It is known that nicotine induces peroxidation of membrane lipids. It also reduces the anti-oxidant capacity of the lungs [40,115]. Since oxidants [33] and nicotine [116] can induce point mutations in DNA (Figure 2), it is possible that the imbalance in the oxidant/antioxidant status of the nicotine-exposed developing lung results in the altered “programming” and consequently the lower glycolytic capacity of the lungs [70], as well as the drastic increase in AMP [75]. This theory is supported by the observation that maternal vitamin C supplementation during pregnancy and lactation prevents the lowering of the glycolytic capacity of the lungs of nicotine-exposed offspring [40] as well as the development of microscopic emphysema (G Maritz, unpublished data). It is therefore plausible that restoration of the oxidant/antioxidant status of the mother and offspring will prevent altered “programming” and thus premature aging of the lungs of the offspring.

It has been demonstrated that *in utero* exposure to nicotine increases DNA methylation and acetylation in the fetus. Nicotine also alters gene methylation in cultured human esophageal squamous epithelial cells [117]. It is therefore plausible that some of the longer term effects of maternal nicotine exposure on the respiratory system of the offspring are due to epigenetic changes. It has been suggested that the rapid induction of insulin resistance in rats exposed to nicotine during gestation and lactation is a reflection of an acute epigenetic response and not a genetic predisposition [118]. It is thus plausible that the effects of maternal nicotine exposure on the metabolism and lung structural integrity of the offspring are due to epigenetic changes rather than changes to the DNA.

## 11. Nicotine and Cell Signaling: Apoptosis and Lung Development

Programmed cell death or apoptosis is an energy-dependent and genetically controlled process [119] that can be induced by a number of molecular tools [120]. Apoptosis occurs in the pulmonary mesenchyme as early as day 14 of gestation in the rat, when branching of conducting airways is the predominant feature. The percentage of cells undergoing apoptosis increases dramatically between 18 and 22 days of gestation and remains elevated in the first day of postnatal life.

During the phase of rapid alveolarisation between postnatal days 4 and 13 in rats, which corresponds to week 36 of gestation in humans [67], interstitial fibroblasts undergo rapid proliferation. Few alveoli are formed after this phase. Between postnatal days 13 and 21 the number of fibroblasts and type II cells decreases. This decrease in fibroblasts and type II cells occurs by means of programmed cell death or apoptosis, which peaks between postnatal days 17 and 19. Apoptosis therefore plays a key role in the thinning of the alveolar septa that occurs after the cessation of alveolarisation [121,122].

Cigarette smoke inhibits the proliferation and migration of human lung fibroblasts and fibroblast-mediated responses and in this way can contribute to the development of emphysema [94]. Nicotine and its metabolite cotinine inhibit apoptosis in fibroblasts [123], but the mechanism is not known. Nicotine is known to exert its effects on many cell types via nicotinic cholinergic receptors. It has been suggested that pediatric, smoking-associated pulmonary diseases and small cell lung carcinoma may be caused by the direct chronic stimulation of an α7-nicotinic acetylcholine receptor-initiated autocrine loop by nicotine and NNK, where NNK is formed from nicotine by nitrosation in the body and during curing of tobacco [124,125]. It is also possible that certain effects of nicotine are not receptor mediated and may operate through unconventional nicotine receptors [123].

There is evidence that nicotine: (a) activates the mitogen-activated protein kinase (MAP) signaling pathway and extracellular signal-regulated kinase (ERK-2), resulting in increased expression of the Bcl-2 protein and inhibition of apotosis, and (b) blocks the inhibition of protein kinase C (PKC) activity in lung cells. Nicotine appears to have no effect on the activities of c-jun NH-2-terminal protein kinase (JNK), c-myc or p28 MAP kinases that are involved in apoptosis. While exposure to nicotine can result in the activation of two major signaling pathways (MAP-kinase and PKC) that are known to inhibit apoptosis, nicotine regulation of MAP and ERK kinase activity is not dependent on PKC. These effects of nicotine occur at concentrations that are generally found in the blood of smokers, and could lead to disruption of the critical balance between cell death and proliferation [58,126]. The inhibition of apoptosis by nicotine may contribute to the slower thinning of the alveolar septa of the lungs of rat pups that were exposed to nicotine during gestation and suckling [127]. Experimental data suggests that nicotine exposure of the fetus and newborn during the phases of rapid cell division may render the lungs more susceptible to the development of cancer [53]. No epidemiologic data are currently available to support this suggestion, although the risk of other forms of cancer may be increased by prenatal exposure to tobacco smoke [128,129].

It has been suggested that *in utero* exposure of pulmonary neuro-endocrine cells to nicotine or NNK may contribute to the development of pediatric lung disorders such as bronchitis and lower respiratory illnesses [53] along with altered pulmonary mechanics in infants and children [130]. The alterations in lung function in monkeys prenatally exposed to nicotine parallel those observed in infants of mothers who smoke during pregnancy [130]. These alterations in lung function could be induced via two mechanisms. The first is a direct effect of released 5-hydroxytryptamine (5-HT) in response to α7 nicotinic receptor stimulation of bronchial and vascular smooth muscle and fibroblast growth; the second is an indirect effect of 5-HT on pulmonary neuro-endocrine cell numbers via activation of a Raf-1/MAP kinase pathway, resulting in yet more cells that can synthesize and release 5-HT. Chronic exposure to nicotine and NNK during early development may therefore up-regulate the α7 nicotinic receptor as well as components of its associated mitogenic signal transduction pathway, thereby increasing the vulnerability of infants to the development of pediatric lung disorders [48].

## 12. Nicotine and Immune Response

The development of immune systems begins during fetal life and proceeds into early neonatal life. This renders them very vulnerable to changes in the environment to which the fetus and neonate are exposed, and may have lasting effects on the immune function of the individual. It has been shown that in adults cigarette smoke is a risk factor for upper respiratory tract infections [131]. Some of the effects of cigarette smoke on the immune system, such as lowered serum IgG and decreased activity and numbers of natural killer cells, may be attributed to nicotine. It was indeed shown in rodents that adolescent nicotine treatment results in impaired T-cell immune responses well after nicotine exposure was terminated. It is therefore plausible that nicotine exposure during early development modulates allergic responses and thereby increases the susceptibility of the offspring to asthma in later life [132].

## 13. Nicotine Replacement Therapy (NRT)

Recently it has been suggested that all pregnant women should stop smoking immediately because if a mother abstains from smoking during the first three months of her pregnancy, the risks to the fetus are the same as those of a fetus of a non-smoking mother [4]. Nicotine replacement therapy (NRT) is prescribed by many health professionals to assist smokers to quit the habit. Various products are currently available to provide nicotine in order to reduce the craving for smoking; these include nicotine containing gums, patches, lozenges, and sprays [133] as well as electronic cigarettes. NRT has been recommended to assist women to quit smoking when they become pregnant. Although NRT is widely prescribed by health professionals as an aid to stop smoking, it is questionable whether nicotine intake during pregnancy and lactation is safe for the fetus and neonate.

The use of NRT is widely promoted because it is often thought that nicotine is not harmful [4]. However, several studies show that nicotine can damage the fetal lungs, heart, and the central nervous system. Nicotine is genotoxic [4,33] and its toxic effects persist in the fetus after administration has stopped [4]. Studies in non-human primates [104] clearly show that nicotine exposure during pregnancy increases the development of α7 nicotinic receptors in cells implicated in lung development. Pulmonary hypoplasia and other abnormalities in pulmonary and bronchial development have been found in the offspring after exposure to nicotine during gestation [48,70,108,111]. It is also evident that nicotine exposure during development suppresses lysyl oxidase activity and this could contribute to the gradual deterioration of the lung parenchyma of the offspring. Furthermore, nicotine induces peroxidation of membrane lipids [84] which changes the oxidant/anti-oxidant status of the lungs of the offspring. This is supported by the decrease in the vitamin C and E content of the lungs of the offspring [134]; these lungs also clearly show a decrease in the levels of the enzymes that catalyze the removal of antioxidants from the lung. Our studies show that the level of superoxide dismutase in the lungs of rats that were prenatally exposed to nicotine remains significantly lower than that of rats not exposed to nicotine (G Maritz, unpublished data). This implies that, apart from its immediate effect in the lungs of those who use NRT, maternal nicotine intake during pregnancy and lactation will have a long-term effect on the maintenance of lung integrity and respiratory health of exposed offspring [42].

## 14. Conclusions

Maternal nicotine exposure during gestation and lactation, and therefore by implication maternal tobacco smoking (and perhaps tobacco chewing), results in a change in the program that controls the development and aging of the lungs of offspring. Changes in the conducting airways and alveoli render the lungs of the offspring more susceptible to disease and reduced lung function. Although it is generally accepted that prevention of maternal smoking during pregnancy and lactation is essential, the considerable evidence of adverse effects on exposed offspring indicates that it is not appropriate to prescribe NRT to pregnant women.

## References

[1] Halima BA, Sarra K, Kais R, Salwa E, Najoua G (2010). Indicators of oxidative stress in weanling and pubertal rats following exposure to nicotine via milk. Hum. Exp. Toxicol.

[2] Ozokutan BH, Ozkan KU, Sari I, Inanc F, Guldur ME, Kilinc M (2005). Effects of maternal nicotine exposure during lactation on breast-fed rat pups. Biol. Neonate.

[3] Oliveira E, Pinheiro CR, Santos-Silva AP, Trevenzoli IH, Abreu-Villaca Y, Nogueira Neto JF, Reis AM, Passos MC, Moura EG, Lisboa PC (2010). Nicotine exposure affects mother’s and pup’s nutritional, biochemical, and hormonal profiles during lactation in rats. J. Endocrinol.

[4] Jiménez Ruiz CA (2006). Tratamiento sustitutivo con nicotina en el embarazo. Arch. Bronconeumol.

[5] Siu EC, Tyndale RF (2007). Non-nicotinic therapies for smoking cessation. Annu. Rev. Pharmacol. Toxicol.

[6] Gonzales D, Rennard SI, Nides M, Oncken C, Azoulay S, Billing CB, Watsky EJ, Gong J, Williams KE, Reeves KR (2006). Varenicline, an alpha4beta2 nicotinic acetylcholine receptor partial agonist, *vs*. sustained-release bupropion and placebo for smoking cessation: A randomized controlled trial. JAMA.

[7] Potts LA, Garwood CL (2007). Varenicline: The newest agent for smoking cessation. Am. J. Health Syst. Pharm.

[8] Hofhuis W, de Jongste JC, Merkus PJ (2003). Adverse health effects of prenatal and postnatal tobacco smoke exposure on children. Arch. Dis. Child.

[9] Lodrup Carlsen KC, Jaakkola JJ, Nafstad P, Carlsen KH (1997). *In utero* exposure to cigarette smoking influences lung function at birth. Eur. Respir. J.

[10] Stocks J, Dezateux C (2003). The effect of parental smoking on lung function and development during infancy. Respirology.

[11] Elliot J, Carroll N, Bosco M, McCrohan M, Robinson P (2001). Increased airway responsiveness and decreased alveolar attachment points following *in utero* smoke exposure in the guinea pig. Am. J. Respir. Crit. Care Med.

[12] Landau LI (2008). Tobacco smoke exposure and tracking of lung function into adult life. Paediatr. Respir. Rev.

[13] Gilliland FD, Berhane K, McConnell R, Gauderman WJ, Vora H, Rappaport EB, Avol E, Peters JM (2000). Maternal smoking during pregnancy, environmental tobacco smoke exposure and childhood lung function. Thorax.

[14] Goksor E, Amark M, Alm B, Gustafsson PM, Wennergren G (2007). The impact of pre- and post-natal smoke exposure on future asthma and bronchial hyper-responsiveness. Acta Paediatr.

[15] Henderson AJ, Newson RB, Rose-Zerilli M, Ring SM, Holloway JW, Shaheen SO (2010). Maternal Nrf2 and gluthathione-S-transferase polymorphisms do not modify associations of prenatal tobacco smoke exposure with asthma and lung function in school-aged children. Thorax.

[16] Upton MN, Smith GD, McConnachie A, Hart CL, Watt GC (2004). Maternal and personal cigarette smoking synergize to increase airflow limitation in adults. Am. J. Respir. Crit. Care Med.

[17] Beyer D, Mitfessel H, Gillissen A (2009). Maternal smoking promotes chronic obstructive lung disease in the offspring as adults. Eur. J. Med. Res.

[18] Brewer BG, Roberts AM, Rowell PP (2004). Short-term distribution of nicotine in the rat lung. Drug Alcohol Depend.

[19] Matta SG, Balfour DJ, Benowitz NL, Boyd RT, Buccafusco JJ, Caggiula AR, Craig CR, Collins AC, Damaj MI, Donny EC, Gardiner PS, Grady SR, Heberlein U, Leonard SS, Levin ED, Lukas RJ, Markou A, Marks MJ, McCallum SE, Parameswaran N, Perkins KA, Picciotto MR, Quik M, Rose JE, Rothenfluh A, Schafer WR, Stolerman IP, Tyndale RF, Wehner JM, Zirger JM (2007). Guidelines on nicotine dose selection for *in vivo* research. Psychopharmacology.

[20] Onuki M, Yokoyama K, Kimura K, Sato H, Nordin RB, Naing L, Morita Y, Sakai T, Kobayashi Y, Araki S (2003). Assessment of urinary cotinine as a marker of nicotine absorption from tobacco leaves: A study on tobacco farmers in Malaysia. J. Occup. Health.

[21] Sastry BV, Chance MB, Hemontolor ME, Goddijn-Wessel TA (1998). Formation and retention of cotinine during placental transfer of nicotine in human placental cotyledon. Pharmacology.

[22] Dahlstrom A, Lundell B, Curvall M, Thapper L (1990). Nicotine and cotinine concentrations in the nursing mother and her infant. Acta Paediatr. Scand.

[23] Luck W, Nau H, Hansen R, Steldinger R (1985). Extent of nicotine and cotinine transfer to the human fetus, placenta and amniotic fluid of smoking mothers. Dev. Pharmacol. Ther.

[24] Benowitz NL, Jacob P (1984). Nicotine and carbon monoxide intake from high- and low-yield cigarettes. Clin. Pharmacol. Ther.

[25] Hukkanen J, Jacob P, Benowitz NL (2005). Metabolism and disposition kinetics of nicotine. Pharmacol. Rev.

[26] Henningfield JE, Stapleton JM, Benowitz NL, Grayson RF, London ED (1993). Higher levels of nicotine in arterial than in venous blood after cigarette smoking. Drug Alcohol Depend.

[27] Suzuki K, Horiguchi T, Comas-Urrutia AC, Mueller-Heubach E, Morishima HO, Adamsons K (1974). Placental transfer and distribution of nicotine in the pregnant rhesus monkey. Am. J. Obstet. Gynecol.

[28] Dempsey DA, Benowitz NL (2001). Risks and benefits of nicotine to aid smoking cessation in pregnancy. Drug Saf.

[29] Frank L, Sosenko IR (1987). Prenatal development of lung antioxidant enzymes in four species. J. Pediatr.

[30] Hayashibe H, Asayama K, Dobashi K, Kato K (1990). Prenatal development of antioxidant enzymes in rat lung, kidney, and heart: Marked increase in immunoreactive superoxide dismutases, glutathione peroxidase, and catalase in the kidney. Pediatr. Res.

[31] Walther FJ, Wade AB, Warburton D, Forman HJ (1991). Ontogeny of antioxidant enzymes in the fetal lamb lung. Exp. Lung Res.

[32] Lambers DS, Clark KE (1996). The maternal and fetal physiologic effects of nicotine. Semin. Perinatol.

[33] Kleinsasser NH, Sassen AW, Semmler MP, Harreus UA, Licht AK, Richter E (2005). The tobacco alkaloid nicotine demonstrates genotoxicity in human tonsillar tissue and lymphocytes. Toxicol. Sci.

[34] Bruin JE, Petre MA, Raha S, Morrison KM, Gerstein HC, Holloway AC (2008). Fetal and neonatal nicotine exposure in Wistar rats causes progressive pancreatic mitochondrial damage and beta cell dysfunction. PLoS One.

[35] Rehan VK, Wang Y, Sugano S, Santos J, Patel S, Sakurai R, Boros LG, Lee WP, Torday JS (2007). *In utero* nicotine exposure alters fetal rat lung alveolar type II cell proliferation, differentiation, and metabolism. Am. J. Physiol. Lung Cell Mol. Physiol.

[36] Guo J, Chu M, Abbeyquaye T, Chen CY (2005). Persistent nicotine treatment potentiates amplification of the dihydrofolate reductase gene in rat lung epithelial cells as a consequence of Ras activation. J. Biol. Chem.

[37] Hartwell LH, Kastan MB (1994). Cell cycle control and cancer. Science.

[38] Noakes PS, Thomas R, Lane C, Mori TA, Barden AE, Devadason SG, Prescott SL (2007). Association of maternal smoking with increased infant oxidative stress at 3 months of age. Thorax.

[39] Orhon FS, Ulukol B, Kahya D, Cengiz B, Baskan S, Tezcan S (2009). The influence of maternal smoking on maternal and newborn oxidant and antioxidant status. Eur. J. Pediatr.

[40] Husain K, Scott BR, Reddy SK, Somani SM (2001). Chronic ethanol and nicotine interaction on rat tissue antioxidant defense system. Alcohol.

[41] Droge W (2002). Free radicals in the physiological control of cell function. Physiol. Rev.

[42] Wallace DC (2005). A mitochondrial paradigm of metabolic and degenerative diseases, aging, and cancer: A dawn for evolutionary medicine. Annu. Rev. Genet.

[43] Zaken V, Kohen R, Ornoy A (2001). Vitamins C and E improve rat embryonic antioxidant defense mechanism in diabetic culture medium. Teratology.

[44] Oliveira E, Moura EG, Santos-Silva AP, Fagundes AT, Rios AS, Abreu-Villaca Y, Nogueira Neto JF, Passos MC, Lisboa PC (2009). Short- and long-term effects of maternal nicotine exposure during lactation on body adiposity, lipid profile, and thyroid function of rat offspring. J. Endocrinol.

[45] Zhao Z, Reece EA (2005). Nicotine-induced embryonic malformations mediated by apoptosis from increasing intracellular calcium and oxidative stress. Birth Defects Res. B Dev. Reprod. Toxicol.

[46] Haustein KO (1999). Cigarette smoking, nicotine and pregnancy. Int. J. Clin. Pharmacol. Ther.

[47] Ornoy A (2007). Embryonic oxidative stress as a mechanism of teratogenesis with special emphasis on diabetic embryopathy. Reprod. Toxicol.

[48] Sekhon HS, Jia Y, Raab R, Kuryatov A, Pankow JF, Whitsett JA, Lindstrom J, Spindel ER (1999). Prenatal nicotine increases pulmonary alpha7 nicotinic receptor expression and alters fetal lung development in monkeys. J. Clin. Invest.

[49] Maneckjee R, Minna JD (1994). Opioids induce while nicotine suppresses apoptosis in human lung cancer cells. Cell Growth Differ.

[50] Maus AD, Pereira EF, Karachunski PI, Horton RM, Navaneetham D, Macklin K, Cortes WS, Albuquerque EX, Conti-Fine BM (1998). Human and rodent bronchial epithelial cells express functional nicotinic acetylcholine receptors. Mol. Pharmacol.

[51] Pontieri FE, Tanda G, Orzi F, Di Chiara G (1996). Effects of nicotine on the nucleus accumbens and similarity to those of addictive drugs. Nature.

[52] Wongtrakool C, Roser-Page S, Rivera HN, Roman J (2007). Nicotine alters lung branching morphogenesis through the alpha7 nicotinic acetylcholine receptor. Am. J. Physiol. Lung Cell. Mol. Physiol.

[53] Sekhon HS, Keller JA, Benowitz NL, Spindel ER (2001). Prenatal nicotine exposure alters pulmonary function in newborn rhesus monkeys. Am. J. Respir. Crit. Care Med.

[54] Proskocil BJ, Sekhon HS, Jia Y, Savchenko V, Blakely RD, Lindstrom J, Spindel ER (2004). Acetylcholine is an autocrine or paracrine hormone synthesized and secreted by airway bronchial epithelial cells. Endocrinology.

[55] Cattaneo MG, D’Atri F, Vicentini LM (1997). Mechanisms of mitogen-activated protein kinase activation by nicotine in small-cell lung carcinoma cells. Biochem. J.

[56] Minna JD (2003). Nicotine exposure and bronchial epithelial cell nicotinic acetylcholine receptor expression in the pathogenesis of lung cancer. J. Clin. Invest.

[57] Chen CY, Liou J, Forman LW, Faller DV (1998). Differential regulation of discrete apoptotic pathways by Ras. J. Biol. Chem.

[58] Heeschen C, Jang JJ, Weis M, Pathak A, Kaji S, Hu RS, Tsao PS, Johnson FL, Cooke JP (2001). Nicotine stimulates angiogenesis and promotes tumor growth and atherosclerosis. Nat. Med.

[59] Macklin KD, Maus AD, Pereira EF, Albuquerque EX, Conti-Fine BM (1998). Human vascular endothelial cells express functional nicotinic acetylcholine receptors. J. Pharmacol. Exp. Ther.

[60] West KA, Brognard J, Clark AS, Linnoila IR, Yang X, Swain SM, Harris C, Belinsky S, Dennis PA (2003). Rapid Akt activation by nicotine and a tobacco carcinogen modulates the phenotype of normal human airway epithelial cells. J. Clin. Invest.

[61] Vogelstein B, Kinzler KW (1992). p53 function and dysfunction. Cell.

[62] O’Neil JJ, Tierney DF (1974). Rat lung metabolism: glucose utilization by isolated perfused lungs and tissue slices. Am. J. Physiol.

[63] Bourbon J, Jost A (1982). Control of glycogen metabolism in the developing fetal lung. Pediatr. Res.

[64] Gilden C, Sevanian A, Tierney DF, Kaplan SA, Barrett CT (1977). Regulation of fetal lung phosphatidyl choline synthesis by cortisol: Role of glycogen and glucose. Pediatr. Res.

[65] Maniscalco WM, Wilson CM, Gross I, Gobran L, Rooney SA, Warshaw JB (1978). Development of glycogen and phospholipid metabolism in fetal and newborn rat lung. Biochim. Biophys. Acta.

[66] Salisbury-Murphy S, Rubinstein D, Beck JC (1966). Lipid metabolism in lung slices. Am. J. Physiol.

[67] Post M, Copland I (2002). Overview of lung development. Acta Pharmacol. Sin.

[68] Maritz GS (1988). Lung glycogen metabolism in suckling rats: A comparative study. Biol. Neonate.

[69] Rhoades RA (1974). Net uptake of glucose, glycerol, and fatty acids by the isolated perfused rat lung. Am. J. Physiol.

[70] Ito T (1999). Differentiation and proliferation of pulmonary neuroendocrine cells. Prog. Histochem. Cytochem.

[71] Maritz G (1986). Pre- and postnatal carbohydrate metabolism of rat lung tissue. The effect of maternal nicotine exposure. Arch. Toxicol.

[72] Ito T, Noguchi Y, Udaka N, Kitamura H, Satoh S (1999). Glucose transporter expression in developing fetal lungs and lung neoplasms. Histol. Histopathol.

[73] Iba MM, Fung J, Pak YW, Thomas PE, Fisher H, Sekowski A, Halladay AK, Wagner GC (1999). Dose-dependent up-regulation of rat pulmonary, renal, and hepatic cytochrome P-450 (CYP) 1A expression by nicotine feeding. Drug Metab. Dispos.

[74] Maritz GS (1987). Maternal nicotine exposure and carbohydrate metabolism of fetal and neonatal lung tissue: response to nicotine withdrawal. Respiration.

[75] Maritz GS, Burger B (1992). The influence of maternal nicotine exposure on neonatal lung carbohydrate metabolism. Cell Biol. Int. Rep.

[76] Kondoh H, Lleonart ME, Gil J, Wang J, Degan P, Peters G, Martinez D, Carnero A, Beach D (2005). Glycolytic enzymes can modulate cellular life span. Cancer Res.

[77] Zwerschke W, Mazurek S, Stockl P, Hutter E, Eigenbrodt E, Jansen-Durr P (2003). Metabolic analysis of senescent human fibroblasts reveals a role for AMP in cellular senescence. Biochem. J.

[78] Kondoh H, Lleonart ME, Bernard D, Gil J (2007). Protection from oxidative stress by enhanced glycolysis; a possible mechanism of cellular immortalization. Histol. Histopathol.

[79] Hukkanen J, Pelkonen O, Raunio H (2001). Expression of xenobiotic-metabolizing enzymes in human pulmonary tissue: possible role in susceptibility for ILD. Eur. Respir. J. Suppl.

[80] Russell MA, Feyerabend C (1978). Cigarette smoking: A dependence on high-nicotine boli. Drug Metab. Rev.

[81] Lee CZ, Royce FH, Denison MS, Pinkerton KE (2000). Effect of *in utero* and postnatal exposure to environmental tobacco smoke on the developmental expression of pulmonary cytochrome P450 monooxygenases. J. Biochem. Mol. Toxicol.

[82] Luck W, Nau H (1984). Nicotine and cotinine concentrations in serum and milk of nursing smokers. Br. J. Clin. Pharmacol.

[83] Raunio H, Hakkola J, Hukkanen J, Lassila A, Paivarinta K, Pelkonen O, Anttila S, Piipari R, Boobis A, Edwards RJ (1999). Expression of xenobiotic-metabolizing CYPs in human pulmonary tissue. Exp. Toxicol. Pathol.

[84] Gamieldien K, Maritz GS (2004). Postnatal expression of cytochrome P450 1A1, 2A3, and 2B1 mRNA in neonatal rat lung: Influence of maternal nicotine exposure. Exp. Lung Res.

[85] Carmella SG, Borukhova A, Akerkar SA, Hecht SS (1997). Analysis of human urine for pyridine-N-oxide metabolites of 4-(methylnitrosamino)-1-(3-pyridyl)-1-butanone, a tobacco-specific lung carcinogen. Cancer Epidemiol. Biomarkers Prev.

[86] Kauffman SL, Burri PH, Weibel ER (1974). The postnatal growth of the rat lung. II. Autoradiography. Anat. Rec.

[87] Massaro GD, Massaro D (1996). Formation of pulmonary alveoli and gas-exchange surface area: Quantitation and regulation. Annu. Rev. Physiol.

[88] Coalson JJ, Winter V, deLemos RA (1995). Decreased alveolarization in baboon survivors with bronchopulmonary dysplasia. Am. J. Respir. Crit. Care Med.

[89] Margraf LR, Tomashefski JF, Bruce MC, Dahms BB (1991). Morphometric analysis of the lung in bronchopulmonary dysplasia. Am. Rev. Respir. Dis.

[90] Kida K, Thurlbeck WM (1980). The effects of beta-aminopropionitrile on the growing rat lung. Am. J. Pathol.

[91] Noguchi A, Samaha H (1991). Developmental changes in tropoelastin gene expression in the rat lung studied by *in situ* hybridization. Am. J. Respir. Cell. Mol. Biol.

[92] Nakamura Y, Romberger DJ, Tate L, Ertl RF, Kawamoto M, Adachi Y, Mio T, Sisson JH, Spurzem JR, Rennard SI (1995). Cigarette smoke inhibits lung fibroblast proliferation and chemotaxis. Am. J. Respir. Crit. Care Med.

[93] Elliot JG, Carroll NG, James AL, Robinson PJ (2003). Airway alveolar attachment points and exposure to cigarette smoke *in utero*. Am. J. Respir. Crit. Care Med.

[94] Holz O, Zuhlke I, Jaksztat E, Muller KC, Welker L, Nakashima M, Diemel KD, Branscheid D, Magnussen H, Jorres RA (2004). Lung fibroblasts from patients with emphysema show a reduced proliferation rate in culture. Eur. Respir. J.

[95] Muller KC, Welker L, Paasch K, Feindt B, Erpenbeck VJ, Hohlfeld JM, Krug N, Nakashima M, Branscheid D, Magnussen H, Jorres RA, Holz O (2006). Lung fibroblasts from patients with emphysema show markers of senescence *in vitro*. Respir. Res.

[96] Verbeken EK, Cauberghs M, Mertens I, Clement J, Lauweryns JM, van de Woestijne KP (1992). The senile lung. Comparison with normal and emphysematous lungs. 1. Structural aspects. Chest.

[97] Naimark A (1977). Nonventilatory functions of the lung. Summary. Am. Rev. Respir. Dis.

[98] Massaro GD, Gail DB, Massaro D (1975). Lung oxygen consumption and mitochondria of alveolar epithelial and endothelial cells. J. Appl. Physiol.

[99] Paul RJ (1983). Functional compartmentalization of oxidative and glycolytic metabolism in vascular smooth muscle. Am. J. Physiol.

[100] Contran RS, Kumar V, Robbins SL, Robbins SL (1989). Cellular injury and adaptation. Pathologic Basis of Disease.

[101] Witsch IH (1976). Proliferation of type II alveolar cells: A review of common responses in toxic lung injury. Toxicology.

[102] Maritz GS, Thomas RA (1994). The influence of maternal nicotine exposure on the interalveolar septal status of neonatal rat lung. Cell Biol. Int.

[103] Berthiaume Y, Voisin G, Dagenais A (2006). The alveolar type I cells: The new knight of the alveolus?. J. Physiol.

[104] Maritz GS, Thomas RA (1995). Maternal nicotine exposure: response of type II pneumocytes of neonatal rat pups. Cell Biol. Int.

[105] Fehrenbach H (2001). Alveolar epithelial type II cell: Defender of the alveolus revisited. Respir. Res.

[106] Hodes RJ (1999). Telomere length, aging, and somatic cell turnover. J. Exp. Med.

[107] Rehan VK, Asotra K, Torday JS (2009). The effects of smoking on the developing lung: Insights from a biologic model for lung development, homeostasis, and repair. Lung.

[108] Maritz G (1997). Maternal nicotine exposure induces microscopic emphysema in neonatal rat lung. Pathophysiology.

[109] Maritz GS, Windvogel S (2003). Is maternal copper supplementation during alveolarization protecting the developing rat lung against the adverse effects of maternal nicotine exposure? A morphometric study. Exp. Lung. Res.

[110] Collins MH, Moessinger AC, Kleinerman J, Bassi J, Rosso P, Collins AM, James LS, Blanc WA (1985). Fetal lung hypoplasia associated with maternal smoking: A morphometric analysis. Pediatr. Res.

[111] Tsuji T, Aoshiba K, Nagai A (2004). Cigarette smoke induces senescence in alveolar epithelial cells. Am. J. Respir. Cell. Mol. Biol.

[112] Maritz GS, Windvogel S (2003). Chronic maternal nicotine exposure during gestation and lactation and the development of the lung parenchyma in the offspring. Response to nicotine withdrawal. Pathophysiology.

[113] Balakrishnan A, Menon VP (2006). Role of hesperidin on nicotine toxicity. Int. J. Pharmacol.

[114] Maritz G, Windvogel S (2005). Does maternal nicotine exposure during different phases of lung development influence the program that regulates the maintenance of lung integrity in the offspring? A comparative morphologic and morphometric study. Trends Comp. Biochem. Physiol.

[115] Kalpana C, Menon VP (2004). Modulatory effects of curcumin on lipid peroxidation and antioxidant status during nicotine-induced toxicity. Pol. J. Pharmacol.

[116] Soma T, Kaganoi J, Kawabe A, Kondo K, Imamura M, Shimada Y (2006). Nicotine induces the fragile histidine triad methylation in human esophageal squamous epithelial cells. Int. J. Cancer.

[117] Holloway AC, Cuu DQ, Morrison KM, Gerstein HC, Tarnopolsky MA (2007). Transgenerational effects of fetal and neonatal exposure to nicotine. Endocrine.

[118] White E (1996). Life, death, and the pursuit of apoptosis. Genes Dev.

[119] Wertz IE, Hanley MR (1996). Diverse molecular provocation of programmed cell death. Trends Biochem. Sci.

[120] Schittny JC, Djonov V, Fine A, Burri PH (1998). Programmed cell death contributes to postnatal lung development. Am. J. Respir. Cell. Mol. Biol.

[121] Bruce MC, Honaker CE, Cross RJ (1999). Lung fibroblasts undergo apoptosis following alveolarization. Am. J. Respir. Cell Mol. Biol.

[122] Wright SC, Zhong J, Zheng H, Larrick JW (1993). Nicotine inhibition of apoptosis suggests a role in tumor promotion. FASEB J.

[123] Fischer S, Spiegelhalder B, Eisenbarth J, Preussmann R (1990). Investigations on the Origin of Tobacco-Specific Nitrosamines in Mainstream Smoke of Cigarettes. Carcinogenesis.

[124] Hecht SS, Hoffmann D (1988). Tobacco-specific nitrosamines, an important group of carcinogens in tobacco and tobacco smoke. Carcinogenesis.

[125] Heusch WL, Maneckjee R (1998). Signalling pathways involved in nicotine regulation of apoptosis of human lung cancer cells. Carcinogenesis.

[126] Maritz GS, Matthews HL, Aalbers J (2000). Maternal copper supplementation protects the neonatal rat lung against the adverse effects of maternal nicotine exposure. Reprod. Fertil. Dev.

[127] Schuller HM, Jull BA, Sheppard BJ, Plummer HK (2000). Interaction of tobacco-specific toxicants with the neuronal alpha(7) nicotinic acetylcholine receptor and its associated mitogenic signal transduction pathway: potential role in lung carcinogenesis and pediatric lung disorders. Eur. J. Pharmacol.

[128] Brooks DR, Mucci LA, Hatch EE, Cnattingius S (2004). Maternal smoking during pregnancy and risk of brain tumors in the offspring. A prospective study of 1.4 million Swedish births. Cancer Cause. Control.

[129] Ng SP, Zelikoff JT (2007). Smoking during pregnancy: subsequent effects on offspring immune competence and disease vulnerability in later life. Reprod. Toxicol.

[130] Argentin G, Cicchetti R (2004). Genotoxic and antiapoptotic effect of nicotine on human gingival fibroblasts. Toxicol. Sci.

[131] Dietert R (2009). Distinguishing environmental causes of immune dysfunction from pediatric triggers of disease. Open Pediat. Med. J.

[132] Mishra NC, Rir-sima-ah J, Langley RJ, Singh SP, Pena-Philippides JC, Koga T, Razani-Boroujerdi S, Hutt J, Campen M, Kim KC, Tesfaigzi Y, Sopori ML (2008). Nicotine primarily suppresses lung Th2 but not goblet cell and muscle cell responses to allergens. J. Immunol.

[133] Benowitz N, Dempsey D (2004). Pharmacotherapy for smoking cessation during pregnancy. Nicotine Tob. Res.

[134] Balakrishnan A, Menon VP (2007). Antioxidant properties of hesperidin in nicotine-induced lung toxicity. Fundam. Clin. Pharmacol.

